# Valorization of Ryegrass Hydrolysates Through Mixotrophic Cultivation of *Desmodesmus subspicatus*: Effects of Carbon and Nitrogen Sources on Biomass and Lipid Composition

**DOI:** 10.3390/md24050149

**Published:** 2026-04-23

**Authors:** Ana Dobrinčić, Klara Komarec, Nenad Marđetko, Antonija Trontel, Mario Novak, Mladen Pavlečić, Vlatka Petravić Tominac, Božidar Šantek

**Affiliations:** Laboratory for Biochemical Engineering, Industrial Microbiology and Malting and Brewing Technology, Department of Biochemical Engineering, Faculty of Food Technology and Biotechnology, University of Zagreb, 10000 Zagreb, Croatianmardetko@pbf.hr (N.M.); atrontel@pbf.hr (A.T.); mnovak@pbf.hr (M.N.); mpavlecic@pbf.hr (M.P.); vpetrav@pbf.hr (V.P.T.); bsantek@pbf.hr (B.Š.)

**Keywords:** *Desmodesmus subspicatus*, mixotrophic cultivation, ryegrass hydrolysate, alkaline and acid pretreatments, nitrogen limitation, lipid productivity, biodiesel, fatty acid profile

## Abstract

Lignocellulosic biomass represents an abundant and renewable carbon source, and its valorization through microalgal cultivation offers a sustainable route to resource-efficient bioprocessing. This study examined the effects of various carbon and nitrogen sources on the growth and lipid metabolism of *Desmodesmus subspicatus*, with a focus on ryegrass enzymatic hydrolysates as an alternative carbon source. Cultures were supplied with glucose, xylose, or arabinose at different concentrations, along with sodium nitrate or yeast extract, under different carbon-to-nitrogen ratios. Additionally, the impacts of alkaline- and acid-pretreated enzymatic ryegrass hydrolysates were evaluated. Growth was assessed by optical density and gravimetric analysis, and fatty acid profiles by gas chromatography. Glucose supplementation enhanced lipid accumulation, yielding fatty acid profiles dominated by C16 and C18 fatty acids, which are favorable for the quality of the produced biodiesel. Nitrogen limitation further promoted lipid accumulation; cultures supplied with sodium nitrate achieved higher total lipid content, while yeast extract favored greater proportions of PUFAs. Alkaline-pretreated ryegrass hydrolysate supported dose-dependent biomass formation reaching approximately 12 g L^−1^ at 50%, whereas the acid-pretreated hydrolysate exhibited inhibitory effects at the same concentration. Scale-up in a 1 L photobioreactor yielded lower biomass but higher lipid content with a fatty acid profile shifted to SFA. These results support ryegrass as a viable alternative carbon source and highlight cultivation parameters that influence growth and lipid quality relevant for biofuel applications.

## 1. Introduction

The majority of carbon-based chemicals (>95%) are still produced from fossil resources [1]. Accelerating climate risks and resource volatility make renewable carbon sources essential for a sustainable bioeconomy. Valorization of low-value residues through clean processes can lower greenhouse gas emissions, reduce dependence on fossil fuels, and create new value chains for fuels, chemicals, and materials [2,3]. Among renewable feedstocks, lignocellulosic biomass is increasingly recognized as a leading source of organic carbon. It consists primarily of cellulose, hemicellulose, and lignin, along with minor constituents such as pectin, nitrogen-containing compounds, and mineral residues [4]. Although highly promising, lignocellulosic biomass, especially in the form of agricultural residues, is often discarded or burned rather than converted into higher-value products such as biofuels, biochemicals, and biopolymers [1]. Compared with conventional sugar-or starch-based raw materials, lignocellulose offers significant economic and environmental advantages due to its lower cost, non-competition with food resources, and broad geographic availability [5]. The key challenge is its complex and recalcitrant structure, a dense matrix of cellulose, hemicellulose, and lignin that resists enzymatic accessibility [4]. Pretreatment is therefore essential to disrupt this complex structure and improve enzymatic saccharification. In general, pretreatment methods can be categorized into physical (milling), chemical (acid, alkaline), physicochemical (steam explosion, ammonia fiber expansion, etc.), and biological kuc. After pretreatment, cellulolytic enzymes convert polysaccharides into fermentable sugars, generating hydrolysates that serve as versatile substrates for microbial fermentation [2]. Among potential raw materials, perennial ryegrass (*Lolium perenne*) is of particular interest owing to its widespread cultivation across Europe, fast growth rate, and high content of fermentable carbohydrates [6].

Microalgae are a promising source of a wide range of valuable bioproducts, including proteins, pigments, and omega-3 fatty acids, as well as lipids and carbohydrates that can be converted into biofuels [7]. Their metabolic profiles vary under autotrophic (CO_2_, light), heterotrophic (organic carbon, dark), or mixotrophic (CO_2_, light, and organic carbon) growth conditions. Recent studies have shown that *Desmodesmus* species can grow under all three conditions, with responses that are strongly species-dependent. In general, mixotrophy and heterotrophy can enhance biomass production compared to autotrophy, while also affecting lipid and protein accumulation [8]. Apart from glucose, the most utilized carbon source, microalgae can also utilize substrates such as glycerol, acetate, and simple sugars (xylose, arabinose). However, these substrates often fail to achieve the level of economic viability required for large-scale industrial cultivation [7]. Therefore, lignocellulosic hydrolysates, rich in mixed sugars and nutrients, offer a cost-effective and resource-efficient alternative. Microalgal growth has previously been demonstrated on hydrolysates derived from sugarcane bagasse [7,9], corn stover [10], wheat straw [11], rice straw [12], and sweet sorghum bagasse [13] or juice [14]. To date, no studies have examined the use of ryegrass enzymatic hydrolysate as a substrate for microalgal cultivation. The existing literature on hydrolysate-based cultivation focuses mainly on a limited number of microalgae species, predominantly *Chlorella*, and to a lesser extent *Desmodesmus* and *Nannochloropsis*. A recent screening of 45 microalgal strains nevertheless identified *Desmodesmus* sp. as one of the few, together with *Chlorococcum* sp. and *Chlamydomonas debaryana*, capable of utilizing corn stover hydrolysate [10].

This research aimed to determine the impact of different carbon sources on the growth and fatty acid profile of *Desmodesmus subspicatus* by cultivating it on various concentrations of glucose, xylose, and arabinose. In addition, the aim was to evaluate the impact of different carbon-to-nitrogen (C/N) ratios, using sodium nitrate and yeast extract as nitrogen sources, on biomass growth and fatty acid profiles. This study also assessed how ryegrass enzymatic hydrolysates, obtained after alkaline and acid pretreatments, impact the *D. subspicatus* growth, cell composition (protein, carbohydrate, chlorophyll), and fatty acid profile. Finally, the results obtained in Erlenmeyer flask cultivations were verified during *D. subspicatus* cultivation in the photobioreactor.

Although *D. subspicatus* is a freshwater species, its use in this study aligns with the broader scope of marine biotechnology, particularly in the context of microalgae-based production of high-value compounds. Lipid metabolism and fatty acid biosynthesis are governed by conserved pathways across freshwater and marine microalgae, making *Desmodesmus* a suitable, cost-effective model system for understanding responses to cultivation conditions relevant to marine species. Furthermore, the use of lignocellulosic hydrolysates as alternative carbon sources represents a sustainable strategy applicable to both freshwater and marine microalgal systems. In addition, several studies have demonstrated that freshwater green microalgae, including species of *Desmodesmus*, exhibit notable tolerance to elevated salinity and can maintain growth under saline conditions. For example, *Desmodesmus* strains have been reported to grow under moderate salt concentrations (up to ~5 g L^−1^ NaCl) [15], while related freshwater species from the same genus have shown the ability to proliferate even at higher salinity levels (up to 10–20 g L^−1^ NaCl) [16], highlighting their potential applicability in saline or marine-like environments.

## 2. Results and Discussion

This study evaluated the growth performance of microalgae *D. subspicatus* under different cultivation conditions. The effect of three carbon sources was tested at concentrations of 5, 10, 25, and 50 g L^−1^ for glucose, and 5, 10, and 25 g L^−1^ for xylose as well as arabinose. The influence of nitrogen source (NaNO_3_ or yeast extract) and varying C/N ratios (10:1, 30:1, and 50:1) was also examined. In addition, the effects of enzymatic ryegrass hydrolysates obtained after alkaline and acid pretreatments were investigated at concentrations of 1%, 5%, 10%, 25%, and 50% (*v*/*v*). All cultivations were carried out in liquid BBM media in Erlenmeyer flasks and in the stirred-tank photobioreactor, as described in Section 3.3 and Section 3.4. The experimental results and their interpretation are presented in the sections below.

### 2.1. Cultivation of D. subspicatus on Different Carbon Sources and Concentrations

#### 2.1.1. Growth of *D. subspicatus* and Carbon Source Utilization

**Figure 1:**
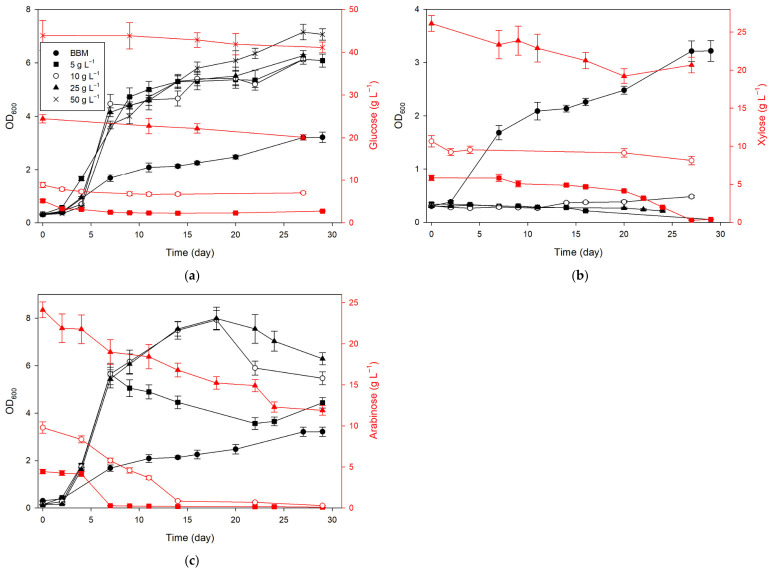
Profiles of optical density (OD_600_; black lines), glucose (**a**), xylose (**b**), and arabinose (**c**) concentrations (red lines) in different media during the cultivation of *Desmodesmus subspicatus*.

The results shown in Figure 1 indicate a difference in the growth of *D. subspicatus* depending on the type and concentration of the carbon source. Both glucose and arabinose supported robust growth, especially at higher concentrations (25 and 50 g L^−1^ for glucose; 10 and 25 g L^−1^ for arabinose), where OD_600_ values exceeded 7 (after dilution) by day 29. Cultures grown on glucose and arabinose showed exponential growth that commenced early, typically between days 4 and 7, with a steady increase throughout the 29-day period. Notably, growth was not inhibited even at 50 g L^−1^ glucose, whereas other studies have reported reduced biomass yields at comparable concentrations due to substrate inhibition [17,18,19]. This discrepancy suggests that the threshold for glucose inhibition is likely species-specific [19]. Gim et al. [17] demonstrated that, among six tested carbon sources, glucose supported the highest biomass yields in various green microalgae, including *Scenedesmus obliquus*, belonging to the same family as the *Desmodesmus* genus. Ren et al. [18] found that *Scenedesmus* sp. strain R-16 reached maximum biomass at 30 g L^−1^ glucose, while both lower and especially higher concentrations (up to 100 g L^−1^) resulted in a noticeable decline in cell growth, indicating that excessive glucose can have an inhibitory effect on algal proliferation. Similarly, Eze et al. [19] reported enhanced growth and lipid accumulation in *D. subspicatus* when cultivated with glucose or glycerol under mixotrophic conditions. Additionally, Saavedra et al. [20] found that *D. subspicatus* could grow in sugarcane vinasse containing arabinose, supporting our results on arabinose utilization.

**Figure A1:**
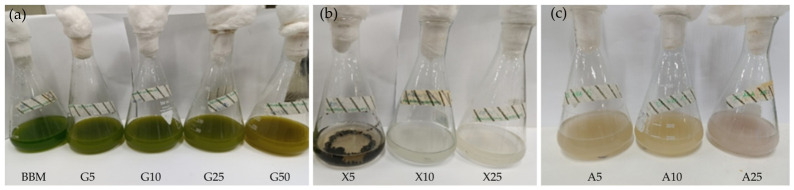
Photos of *Desmodesmus subspicatus* cultivated at different concentrations of (**a**) glucose, (**b**) xylose, and (**c**) arabinose at the end of the cultivation period (Day 29).

In contrast, growth on xylose was minimal and did not differ from the control. OD_600_ values remained below 0.5 in all xylose experiments, with no clear growth trend over time. Notably, the slightly higher biomass increase on xylose was recorded at a concentration of 10 g L^−1^. However, microalgae grown on xylose began to form spherical aggregates (Figure A1) after 14 days, which may have negatively affected the accuracy of optical density measurements and led to the lower values observed. Previous studies on *Scenedesmus* species under mixotrophic conditions have shown that optimal growth and lipid accumulation occur at lower xylose concentrations, typically around 4 g L^−1^, while higher concentrations can inhibit growth [21,22]. Similarly, Sartori et al. [9] reported that *Desmodesmus* sp. exhibited optimal growth at 4 g L^−1^ xylose, with reduced growth observed at concentrations above 6 g L^−1^. These findings suggest that elevated xylose concentrations may have an inhibitory effect on microalgal growth, which could explain the limited biomass increase observed in our study.

Chai et al. [23] have reported that the addition of xylose, even at the lowest concentration, completely inhibited pigment synthesis, resulting in pigment content in *Chlorella sorokiniana* under light conditions comparable to that in cultures grown in the dark. Results presented in Figure A1 also show this phenomenon. It can be observed that *D. subspicatus* cultivated on glucose retained a green color while the samples cultivated on xylose exhibited noticeable discoloration, except for sample X5, in which dark green spherical aggregates were observed [23]. Furthermore, the addition of more than 2 g L^−1^ xylose caused *S. obliquus* to lose its green color within just one day [21]. In addition, Leite et al. [24] demonstrated in *Chlorella* and *Scenedesmus* strains that the decrease in chlorophyll content induced by xylose occurred both under mixotrophic and heterotrophic conditions, indicating that this effect was not light dependent but rather linked to the presence of xylose itself. Xylose may reduce chlorophyll content by inhibiting photosynthetic activity and disrupting the photosynthetic carbon cycle, thereby limiting the availability of energy and NADPH required for pigment biosynthesis [23].

For comparison, in the control culture on BBM medium without any added carbon source, an OD_600_ value of approximately 3.2 was reached after 29 days of cultivation, indicating that while *D. subspicatus* is capable of photoautotrophic growth, the presence of glucose or arabinose considerably enhances biomass production under mixotrophic conditions.

Table 1 presents the percentage of carbon source utilization (∆S), while Figure 1 shows the profiles in glucose, xylose, and arabinose concentrations during the cultivation of *D. subspicatus*. The efficiency of carbon source utilization by *D. subspicatus* varied notably depending on the type and concentration of the substrate. The highest ∆S was observed at the lowest tested concentrations (5 g L^−1^) for all three sugars: 47.29 ± 1.42%, 93.47 ± 2.68%, and 97.64 ± 2.54% for glucose, xylose, and arabinose, respectively. As the concentration increased, ∆S generally declined, particularly for glucose, where only 6.34 ± 0.33% was consumed at 50 g L^−1^.

Notably, the relationship between substrate consumption and biomass production was not always linear, especially in cultures with xylose. Sample X5 showed very high xylose consumption (93.47 ± 2.68%), yet the corresponding OD_600_ value remained low. This discrepancy may be partially explained by the formation of spherical aggregates in this culture, which likely interfered with optical density measurements. In addition to this physical factor, metabolic factors likely contributed to the observed patterns. Sartori et al. [9] reported similar findings for *Desmodesmus* sp., in which rapid xylose consumption occurred under heterotrophic conditions, with no significant cell growth. They proposed that xylose may have been redirected toward the synthesis of secondary metabolites, possibly as part of a metabolic adaptation to integrate xylose into alternative pathways. Conversely, the efficient uptake of pentoses, such as xylose and arabinose, at lower concentrations suggests that *D. subspicatus* possesses active, potentially less-regulated transport and metabolic systems for these sugars [9].

#### 2.1.2. Fatty Acid Composition of *D. subspicatus*

The fatty acid composition of *D. subspicatus* cultivated under different carbon sources is presented in Table 2. Across all experiments, the most abundant fatty acids were palmitic acid (C16:0), oleic acid (C18:1), and linoleic acid (C18:2), with α-linolenic acid (C18:3) also contributing significantly in several samples. Similar fatty acid patterns have been reported in *Desmodesmus* and *Scenedesmus* strains, where C16:0 and C18:1 are frequently the most abundant, followed by C18:2 and C18:3 depending on species and cultivation conditions [25,26,27,28,29]. The fatty acid profile is an important indicator of biodiesel suitability. C16–C18 fatty acids enhance the combustion performance of biodiesel, while a higher proportion of unsaturated fatty acids improves its low-temperature flow properties [29]. High proportions of monounsaturated fatty acids (MUFA) balance oxidative stability with cold flow properties and thus contribute to overall fuel performance [30]. In all samples in this study, C16–C18 fatty acids accounted for 88.7%–94.59% of the total fatty acids, values similar to or slightly higher than those reported in the literature [26,27,28,29].

The control sample in BBM displayed a balanced lipid profile, with 31.53 ± 0.15% saturated fatty acids (SFA), 31.47 ± 0.10% MUFA, and 37 ± 0.21% polyunsaturated fatty acids (PUFA). Glucose supplementation significantly shifted the lipid profile toward higher SFA content, peaking at 42.38 ± 0.30% in G10, primarily due to the dominance of C16:0. MUFA content was moderately reduced, decreasing from 31.47 ± 0.10% in BBM to 22.38 ± 0.19%–27.48 ± 0.16% across different glucose concentrations. Within the PUFA group, C18:2 increased in all glucose-supplemented cultures, whereas C18:3 declined from 21.69 ± 0.78% in BBM to 8.20 ± 0.38% at G50, resulting in an overall decrease in PUFA content with rising glucose concentrations. Similarly, a study on *Auxenochlorella* protothecoides found that glucose supplementation caused significant reductions in PUFAs, from 62.52% to 33.65% (especially a drop in C18:3), and an increase in the SFA and MUFA fractions [31]. Likewise, Yun et al. [32] reported that mixotrophic cultivation of *Chlorella* strains with glucose enhanced the proportion of SFA by approximately 5–10% and MUFA by about 20%, while reducing the PUFA fraction, compared to photoautotrophic growth.

Due to the extremely low biomass concentration in cultures of *D. subspicatus* grown with xylose as the carbon source, it was not possible to obtain sufficient biomass for fatty acid analysis, even after pooling biomass from multiple flasks.

In cultures supplemented with 5 g L^−1^ arabinose, SFA and MUFA content were both reduced, while PUFA content was markedly increased primarily due to a high proportion of C18:2, which reached 42.01 ± 0.95%. In contrast, in cultures grown with 10 g L^−1^ and 25 g L^−1^ arabinose, SFA levels nearly doubled compared to BBM, with a pronounced increase in stearic acid (C18:0), which reached 23.57 ± 0.76% and 26.71 ± 0.31%, respectively. MUFA content remained comparable to that of the control, with C18:1 remaining a major component. However, PUFA content declined drastically in these conditions, particularly due to a strong reduction in C18:3, which fell to 4.83 ± 0.26% and 3.61 ± 0.34%, compared to 21.69 ± 0.78% in BBM. To the best of our knowledge, very few studies have examined the effect of arabinose on microalgal fatty acid composition. However, some studies suggest that arabinose and xylose are primarily channeled into carbohydrate rather than lipid biosynthesis [33]. Furthermore, the limited utilization of arabinose may elicit a stress response comparable to nutrient deprivation (e.g., nitrogen), as observed with xylose by Leite et al. [24]. Such stress often reduces the activity of desaturase enzymes that produce unsaturated fatty acids, leading to a shift toward more SFA [34]. In this way, limited utilization of arabinose can impair desaturation processes, favoring the accumulation of SFA at the expense of PUFA.

Lipid content (W_L_) of *D. subspicatus* biomass increased under glucose supplementation, reaching up to 45.6 ± 0.13% at 5 g L^−1^ and remaining above 24% across all cultivations with glucose, compared to only 6.45 ± 0.15% in the BBM control. In contrast, arabinose supplementation resulted in more modest lipid accumulation (13.31 ± 0.27%–19.04 ± 0.23%), indicating that glucose was a far more effective carbon source for enhancing lipid storage. Similarly, among three monosaccharides (fructose, maltose, glucose), three organic acids (acetate, propionate, butyrate), and one disaccharide (sucrose), glucose showed the highest total lipid content (43.4%) [19]. Furthermore, when glucose concentration was varied from 5 to 100 g L^−1^ (5, 10, 30, 50, 70, 100 g L^−1^), the highest total lipid content (43.4%) was observed at 10 g L^−1^, while higher glucose concentrations reduced total lipid content [19].

### 2.2. Cultivation of D. subspicatus on Different Nitrogen Sources and Carbon to Nitrogen Ratios

#### 2.2.1. Growth of *D. subspicatus* and Carbon Source Utilization

**Figure 2:**
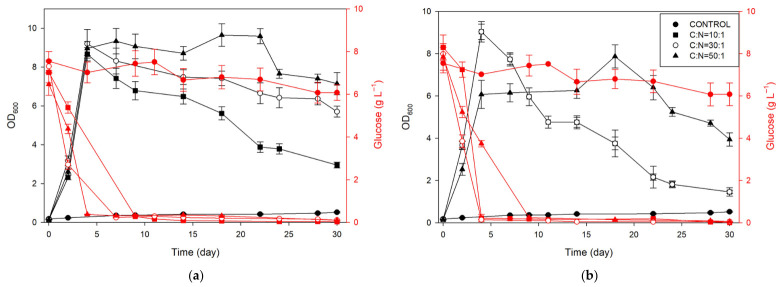
Profiles of optical density (OD_600_; black lines) and changes in glucose concentration (red lines) during the cultivation of *Desmodesmus subspicatus* on (**a**) sodium nitrate and (**b**) yeast extract as nitrogen source.

**Figure A2:**
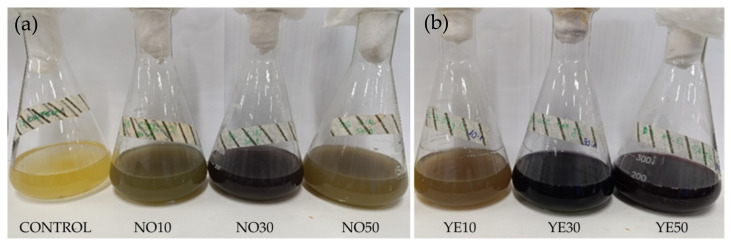
Photos of *Desmodesmus subspicatus* cultivated on (**a**) sodium nitrate and (**b**) yeast extract as nitrogen source at the end of the cultivation period (Day 29).

The results of *D. subspicatus* cultivation under different nitrogen sources and C/N ratios are presented in Figure 2. Growth was strongly influenced by both the nitrogen source and the C/N ratio. Compared to the nitrogen-free control, all nitrogen-supplemented cultures showed significantly enhanced biomass accumulation. Among the tested C/N ratios, 30:1 and 50:1 consistently supported the highest optical density (OD_600_) values, particularly in the NaNO_3_ group. This more gradual and sustained growth under NaNO_3_, especially at C/N 50:1, suggests that *D. subspicatus* maintains biomass more effectively under steady nitrate availability. Interestingly, although all NaNO_3_ additions supported stable growth throughout the cultivation period, cultures supplemented with yeast extract (YE) showed a more rapid increase in biomass during the early growth phase (days 2–7). However, this initial boost was followed by an earlier and sharper decline, particularly in YE10. This suggests that YE may promote faster but less stable growth compared to NaNO_3_, possibly due to rapid nutrient depletion or metabolic imbalances over time. In addition, YE, as a complex mixture of amino acids, peptides, salts, and phosphates that can slightly acidify the medium, may have altered the culture pH and thereby contributed to the observed growth patterns. Furthermore, at a lower C/N ratio (10:1), growth was notably reduced across both nitrogen sources, suggesting that excess nitrogen or carbon limitation may have restricted biomass accumulation. These findings emphasize the importance of carefully selecting nitrogen sources and optimizing the C/N ratio to achieve rapid, sustained microalgal growth (Figure A2).

The observed differences in microalgal growth across nitrogen sources can be attributed to their distinct biochemical compositions. While NaNO_3_ provides nitrate as a pure inorganic nitrogen source, YE contains a complex mix of organic nitrogen compounds, amino acids, vitamins, and other growth-promoting factors that may stimulate cell metabolism and support mixotrophic growth [35]. In a comparative study by Kim et al. [35], YE led to the highest *Tetraselmis* sp. cell concentration after only 2 days of cultivation among nine nitrogen sources tested (NaNO_3_, KNO_3_, NH_4_NO_3_, NH_4_HCO_3_, NH_4_Cl, CH_3_COONH_4_, urea, glycine, and YE), likely due to its dual role as a nitrogen and organic carbon provider. Similarly, *Scenedesmus acutus* demonstrated a strong ability to utilize YE as the sole nitrogen source, achieving high biomass yields even in the absence of nitrate [36]. In contrast, species like *Chlorella* and *Nannochloropsis* were less effective at assimilating nitrogen from YE, indicating that the capacity to exploit complex organic nitrogen sources is highly species-dependent [36].

Glucose consumption was monitored over time in all experimental setups, each supplemented with 10 g L^−1^ of glucose, and results are presented in Figure 2, while carbon source consumption (∆S) is presented in Table 3. In all experiments with a nitrogen source, almost complete glucose depletion occurred, whereas in the control (CONTROL), less than 20% of the initial glucose was consumed throughout the cultivation period. The dynamics of glucose consumption varied depending on both the type of nitrogen source and the C/N ratio. In samples with sodium nitrate, higher C/N ratios were associated with faster glucose consumption: in NO50, glucose was almost entirely consumed by day 4; in NO30, by day 7; and in NO10, by day 9. A similar trend was observed in samples with yeast extract, although with slightly different dynamics. In YE10 and YE30, nearly all glucose was consumed by day 4, whereas in YE50, significant glucose remained until day 9. These results indicate that not only the presence of a nitrogen source, but also its type and the C/N ratio, strongly influence the rate of glucose utilization, with higher C/N ratios generally promoting faster consumption.

#### 2.2.2. Fatty Acid Composition of *D. subspicatus*

The fatty acid composition of *D. subspicatus* cultivated under different nitrogen sources and C/N ratios is presented in Table 4. Across all experiments, the predominant fatty acids were C16:0, C18:1, C18:2, and C18:3, a distribution largely consistent with the patterns observed on algal biomass cultivated with different carbon sources (see Section 2.1.2). In the nitrogen-free control sample, PUFA were the most abundant (52.18 ± 0.17%), followed by SFA (28.81 ± 0.24%) and MUFA (19.01 ± 0.23%). Compared to the control, most experiments showed a general decrease in C16:0 and C18:3, accompanied by an increase in C18:1 and C18:2. These changes are in line with the previously discussed notion that nitrogen limitation affects desaturase activity, resulting in reduced synthesis of unsaturated fatty acids and a relative enrichment in saturated fractions [37].

Similarly, in *Desmodesmus quadricaudatus*, SFA content was higher in nitrogen-free medium (66.92%) compared to sodium nitrate-supplemented medium (51.62%), whereas the proportion of unsaturated fatty acids increased with nitrate addition [38]. An opposite trend was reported for *Tetraselmis* sp., where yeast extract supplementation reduced PUFA (from 43.33% to 33.29%) and increased SFA (from 42.57% to 52.37%), while MUFA remained unchanged [35]. In *Nannochloropsis oceanica*, nitrogen source strongly influenced the fatty acid profile: sodium nitrate supported higher proportions of C18:2 and C16:0, whereas urea shifted the profile toward C22:0 and C18:3 [39].

Furthermore, PUFA content in *D. subspicatus* was consistently higher or comparable in cultures supplemented with yeast extract. In contrast, all samples containing sodium nitrate showed lower PUFA levels than the control. These patterns indicate that yeast extract favors PUFA accumulation, while sodium nitrate supplementation tends to shift the profile toward higher MUFA and SFA fractions. MUFA levels increased in all samples, while SFA displayed a noticeable decrease, specifically at the 30:1 C/N ratio, with both nitrogen sources. In fact, the highest SFA proportions were observed at the 10:1 ratio and the lowest at 30:1. In contrast, PUFA showed the opposite trend, reaching maximum levels at 30:1 and the lowest at 10:1. Comparable effects have also been reported in other species: in *Chlorella* sp., increasing the initial C/N ratio from 29:1 to 95:1 raised C18:1 from 11–20% to 23–24% while reducing PUFA [40], and in *S. obliquus*, nitrate supplementation increased C16:0 from 31.7% to 51.9% and decreased C18:1 as well as the overall unsaturated fraction [30].

Lipid content in biomass (W_L_) (Table 4) of the control sample was 5.54 ± 0.21%. Meanwhile, supplementation with sodium nitrate showed a clear increase with rising C/N ratio, from only 1.59 ± 0.24% at 10:1 up to 17.66 ± 0.26% at 50:1. A similar trend was observed with yeast extract, where lipid content was lowest at 10:1 (1.28 ± 0.23%) and increased to 11.39 ± 0.20% and 13.84 ± 0.15% at 30:1 and 50:1, respectively. These results indicate that stronger nitrogen limitation, at higher C/N ratios, promoted lipid accumulation regardless of the nitrogen source, although absolute values remained lower with yeast extract than with sodium nitrate. Similarly, among eight nitrogen sources (beef extract, urea, peptone, ammonium chloride, sodium nitrate, L-cysteine, yeast extract, and sodium glutamate), the maximum total lipid content of 44.8% was obtained with peptone, closely followed by sodium nitrate. Meanwhile, yeast extract resulted in much lower lipid content in *Scenedesmus* sp. [19]. Furthermore, the same research reported that total lipid content decreased from 52.6% to 13.7% when sodium nitrate concentration increased from 0.2 to 1.0 g L^−1^ [19]. Generally, nitrogen limitation is an effective technique used to increase cellular lipid content in microalgae by shifting metabolism toward the accumulation of triacylglycerols stored in the cytoplasm of microalgae as a source of carbon and energy [41]. Moreover, under nitrogen-limiting conditions, thylakoid membranes are reduced, and lipases (acyl hydrolases) drive phospholipid hydrolysis, elevating intracellular fatty acyl-CoA pools that feed triacylglycerol synthesis [41].

### 2.3. Cultivation of D. subspicatus on Media with Ryegrass Enzymatic Hydrolysate

#### 2.3.1. Composition of Ryegrass Enzymatic Hydrolysate

Pretreatment methods are known to strongly affect the composition of lignocellulosic hydrolysates. Acid pretreatment often promotes hemicellulose solubilization, resulting in higher proportions of pentoses such as xylose and arabinose, but simultaneously generates more inhibitory compounds, including acetic acid, furfural, and 5-hydroxymethylfurfural (HMF) [42]. In contrast, alkaline pretreatment primarily removes lignin and preserves cellulose, thereby usually enhancing enzymatic accessibility and leading to higher glucose recovery with fewer degradation by-products [43]. The results obtained in this study of ryegrass enzymatic hydrolysate (Table 5) are generally consistent with the literature: glucose and xylose concentrations were higher after alkaline pretreatment [44]. Meanwhile, arabinose levels were comparable across treatments. The detection of acetic acid in both hydrolysates, with slightly higher levels in the acid-pretreated sample, is consistent with previous observations that acetic acid is produced by the release of the acetyl group during hemicellulose hydrolysis, which is more pronounced under acidic conditions [43].

#### 2.3.2. Growth of *D. subspicatus* and Carbon Source Utilization

**Figure A3:**
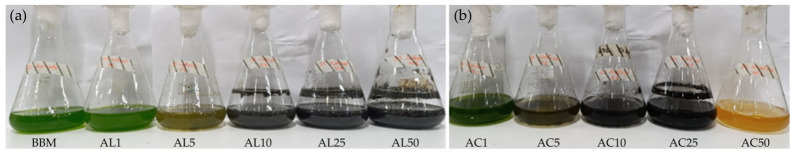
Photos of *Desmodesmus subspicatus* cultivated on (**a**) alkaline-pretreated and (**b**) acid-pretreated ryegrass enzymatic hydrolysate at the end of the cultivation period (Day 15).

The growth of *D. subspicatus* on media with ryegrass hydrolysates was initially monitored by measuring optical density at 600 nm. However, reliable readings could not be obtained due to the rapid increase in culture viscosity, which prevented accurate spectrophotometric measurements (Figure A3). This limitation was particularly pronounced in higher hydrolysate concentrations, where the cultures became too dense for spectrophotometric determination. The increased viscosity is likely a result of extracellular polysaccharide production, triggered by the high organic carbon content and potential nutrient imbalances [45] in ryegrass hydrolysates. Consequently, biomass growth was evaluated gravimetrically, as presented in Table 6.

The results revealed pronounced differences between alkaline- and acid-pretreated hydrolysates. Cultures grown on alkaline hydrolysates showed a clear concentration-dependent increase in biomass, reaching a maximum of 12.03 ± 0.14 g L^−1^ at 50% hydrolysate (AL50), which represents almost a 30-fold increase compared to the control (BBM, 0.41 ± 0.09 g L^−1^). In contrast, cultures on acid hydrolysates demonstrated lower biomass yields overall, with a peak of 4.40 ± 0.26 g L^−1^ at 25% hydrolysate (AC25), followed by a strong inhibition at the highest concentration (AC50, 0.35 ± 0.09 g L^−1^), suggesting toxic effects of inhibitory compounds released during acid pretreatment. A similar trend was observed for biomass productivity, with the highest value also recorded in AL50 and strongly reduced productivity at AC50 (Table 6). These observations are consistent with the visual appearance of the cultures (Figure A3), where alkaline hydrolysates supported robust and dense biomass accumulation. Meanwhile, acid hydrolysates at higher concentrations resulted in darker coloration but lower growth.

Similar trends have been observed in other studies, where lignocellulosic hydrolysates supported higher algal growth compared to conventional media or pure sugars. For instance, *Nannochloropsis* sp. BR2 cultivated mixotrophically on sugarcane bagasse hydrolysate achieved up to 19% higher biomass productivity than in standard F/2 medium [7], and a similar improvement was reported for *Chlorella vulgaris* on wheat straw hydrolysate, where mixotrophic cultivation resulted in higher productivity compared to BG11 medium [11]. In addition, enzymatic hydrolysates of sweet sorghum juice increased *Chlorella protothecoide* biomass production by 37.8% compared with glucose, whereas acid hydrolysates of the same substrate reduced biomass by 10.8% [14]. Likewise, studies with sweet sorghum bagasse [13], rice straw [12], and sugarcane bagasse [46] consistently reported enhanced biomass yields/productivity in hydrolysates relative to pure sugars. Collectively, these findings suggest that, beyond supplying fermentable carbohydrates, hydrolysates provide additional nutrients and growth-promoting factors—such as phosphorus, calcium, vitamins, or amino acids—that stimulate microalgal proliferation [13].

**Figure 3:**
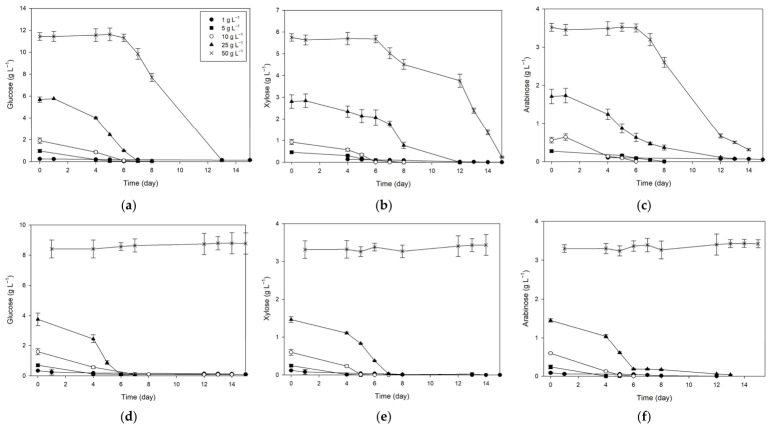
Profiles of glucose (**a**), xylose (**b**), and arabinose (**c**) concentration in media with alkaline-pretreated ryegrass hydrolysate content. Profiles of glucose (**d**), xylose (**e**), and arabinose (**f**) concentration in media with acid-pretreated ryegrass hydrolysate content.

UPLC analysis of sugar consumption (Figure 3) and the calculated carbon source consumption (∆S, Table 7) during the cultivation of *D. subspicatus* on ryegrass hydrolysates can be better understood when considering the initial hydrolysate composition (see Section 2.3.1). Alkaline pretreatment resulted in higher glucose and xylose concentrations and lower acetic acid levels compared to the acid hydrolysate, while arabinose was present at similar levels. These compositional differences may have contributed to the more uniform uptake dynamics observed in alkaline hydrolysates. At lower concentrations (≤10%) of alkaline hydrolysates, sugar utilization occurred rapidly, with most substrates depleted within 4–6 days, although in sample AL1, glucose and arabinose remained only partially consumed (<50%) despite the observed biomass growth. Increasing the concentration to 25% prolonged the uptake period to 7–12 days, while at 50%, complete depletion was achieved only after 12–15 days. A similar pattern was observed in acid hydrolysates, where low and moderate concentrations supported efficient consumption within 4–6 days, with uptake at 25% extending up to 12 days. However, the highest acid hydrolysate concentration (50%) almost completely inhibited sugar consumption (∆S < 2%) in *D. subspicatus,* likely due to elevated acetic acid and/or other inhibitory compounds, consistent with the minimal biomass growth previously observed at this concentration. Overall, apart from this strongly inhibitory effect in the 50% acid hydrolysate, sugar utilization patterns were largely comparable between the two pretreatments. Both alkaline and acid hydrolysates were consumed with similar kinetics and reached comparable final ∆S values, indicating that the pretreatment method had only a minor impact on overall utilization efficiency.

#### 2.3.3. Fatty Acid Composition of *D. subspicatus*

Total lipid content in biomass, lipid productivity (Pr_L_), and fatty acid composition (Table 8; control BBM values are presented in Table 2) of *D. subspicatus* biomass varied notably depending on the type and concentration of ryegrass hydrolysate. Cultures cultivated with alkaline hydrolysates showed a strong increase in lipid content in their biomass, with values more than doubling at lower concentrations and reaching 29.05 ± 0.09% at 50%, almost five times higher than the control (6.45 ± 0.15%). In contrast, acid hydrolysates led to a moderate increase at 5% (11.84 ± 0.10%), but lipid content progressively declined at higher concentrations, approaching the control level at 25%. This suggests that alkaline pretreatment is more effective in promoting lipid accumulation. Consistent with this, lipid productivity increased sharply with alkaline-pretreated ryegrass hydrolysate in a clear dose-dependent manner, reaching values far above the control (BBM 0.18 ± 0.04 g L^−1^ day^−1^). Acid-pretreated hydrolysate also resulted in lipid productivities above the BBM control, but they remained below those of the alkaline treatments at comparable doses, with the higher lipid productivities arising primarily from higher biomass accumulation rather than elevated cellular lipid fractions. Across studies, several hydrolysates increased lipid content and lipid productivity relative to glucose: in *C. protothecoides*, sugarcane bagasse hydrolysate increased lipids from 21.5% to 34% and lipid productivity from 100.62 mg L^−1^ day^−1^ to 493 mg L^−1^ day^−1^ [43], and in *C. pyrenoidosa*, rice straw hydrolysate raised lipids from 50.3% to 56.3% and lipid productivity from 0.19 g L^−1^ day^−1^ to 0.62 g L^−1^ day^−1^ [12]. By contrast, no significant differences were observed when enzymatic sweet sorghum juice hydrolysate replaced glucose in *C. protothecoides* (52.5% vs. 53.3%) [14], or when sugarcane bagasse hydrolysate replaced xylose in *Desmodesmus* sp. [9].

Compared to the BBM control, the proportion of SFA increased in all hydrolysate cultivated biomass except AC25, while MUFA generally decreased, with the sole exception of AL25. PUFA levels exceeded the control only in AL1 and AC25, whereas in other samples they remained lower. Consistent with earlier observations, the fatty acid profile was dominated by C16:0, C18:1, and C18:2. Across all hydrolysate-supplemented cultures, C18:1 and C18:2 were consistently elevated, whereas C18:3 declined relative to the control. Similarly, C18:1 and C18:2 were increased, and C18:3 decreased in mixotrophic cultivation of *Chlorella* sp., compared to autotrophic cultivation [47]. In *C. vulgaris*, supplying sweet sorghum bagasse hydrolysate as an organic carbon source increased lipid productivity, the proportions of C18:1 and C18:3, and, particularly, MUFA content, relative to the BBM control [13].

#### 2.3.4. The Content of Other Constituents of *D. subspicatus*

Protein content in *D. subspicatus* biomass, cultivated on ryegrass hydrolysates, varied markedly across experiments (Table 9). The highest value was observed in the control sample, while both alkaline and acid hydrolysates generally supported lower protein accumulation. Among alkaline hydrolysates, protein levels decreased with increasing hydrolysate concentration, reaching the lowest values at 50% (5.79 ± 0.05%). A similar trend was seen with acid hydrolysates, although moderate concentrations (25%) supported a partial recovery (10.51 ± 0.15%). These results suggest that hydrolysate supplementation, particularly at higher concentrations, may impose stress conditions (e.g., nitrogen limitation and/or the presence of inhibitory compounds) that limit protein biosynthesis in the algal biomass. A similar decrease in protein content following hydrolysate supplementation has been reported by Arora and Philippidis [13], who observed 45% reduction in *C. vulgaris* protein content when cultivated on sweet sorghum bagasse hydrolysate under mixotrophic and heterotrophic conditions, mainly due to nitrate depletion. In contrast, other studies have demonstrated the opposite effect, with hydrolysates enhancing protein contents. For example, wheat straw hydrolysates increased protein content in *C. vulgaris* by 30% relative to autotrophic growth [11], while *Nannochloropsis* sp. grown on sugarcane bagasse hydrolysates showed 7–10% higher protein content compared to standard medium [7]. Moreover, cultivation of *Desmodesmus* sp. on corn stover hydrolysates resulted in increased amino acid content compared to growth in BBM medium without added carbon, reflecting an enhancement of the protein fraction in the biomass [10].

As shown in Table 9, carbohydrate content increased above the BBM control (24.71 ± 0.11%) in all cultivations with the addition of hydrolysate, with the strongest increases at lower hydrolysate concentrations. Accordingly, 1% alkaline ryegrass hydrolysate gave the highest carbohydrate content (42.50 ± 0.29%), which declined to approximately 28–33% at 10–50%. With acid ryegrass hydrolysate, carbohydrate content was likewise elevated at 1–5% (41.08 ± 0.21%–38.98 ± 0.15%) and then plateaued around 30.8% at 10–25%. These findings are consistent with the literature, which shows that mixotrophic growth promotes the accumulation of storage carbohydrates, such as starch. Similar trends were reported for *Desmodesmus/Scenedesmus* and *Chlorella*, where mixotrophic cultivation resulted in higher carbohydrate content compared to autotrophic and/or heterotrophic growth [9,48,49].

Total chlorophyll content (Table 9) of *D. subspicatus* showed a strong reduction in comparison with the control (BBM, 10.95 ± 0.13 mg g^−1^) when ryegrass hydrolysates were added to the cultivation medium. At lower hydrolysate levels (1% and 5%), both alkaline- and acid-pretreated hydrolysates reduced chlorophyll content by approximately 40 to 60%. At higher hydrolysate concentrations (10%, 25%, and 50%), the decrease was even more intense, from 80 to over 90%, indicating severe pigment depletion under these conditions. Similar findings were observed by Manzoor et al. [7], wherein the chlorophyll content of *Nannochloropsis* sp. cultivated on sugarcane bagasse hydrolysate was approximately 10% lower than in photoautotrophic cultivation without a carbon source. Furthermore, the content of lutein, trans-betacarotene, cis-betacarotene, and violaxanthin were 100%, 58%, 61%, and 44% lower than their respective values obtained in photoautotrophic cultivation [7]. As explained by Manzoor et al. [7], the presence of organic carbon in the medium can inhibit photosynthetic apparatus formation, leading to reduced pigment content observed under these conditions.

Microalgae can synthesize diverse polyphenol compounds, including quercetin, gallic acid, vanillic acid, 4-hydroxybenzoic acid, benzoic acid, caffeic acid, and chlorogenic acid [50]. These bioactive compounds are linked to antioxidant, anti-inflammatory, antimicrobial, anti-hypertensive, anti-arthritic, and cardioprotective effects [25]. The content of total polyphenols in *D. subspicatus* biomass was generally very low, remaining below 4 mg GAE g^−1^ in all experiments (Table 9). The control sample (BBM) showed the highest phenolic content (3.23 ± 0.03 mg GAE g^−1^), whereas all samples with hydrolysates resulted in lower values. These results fall toward the lower end of the 0.38–7.7 mg GAE g^−1^ range reported for other *Scenedesmus* species [28,50,51].

### 2.4. Cultivation of D. subspicatus on Media with Ryegrass Enzymatic Hydrolysate in the Stirred-Tank Photobioreactor

#### 2.4.1. Growth of *D. subspicatus* and Carbon Source Utilization

**Figure 4:**
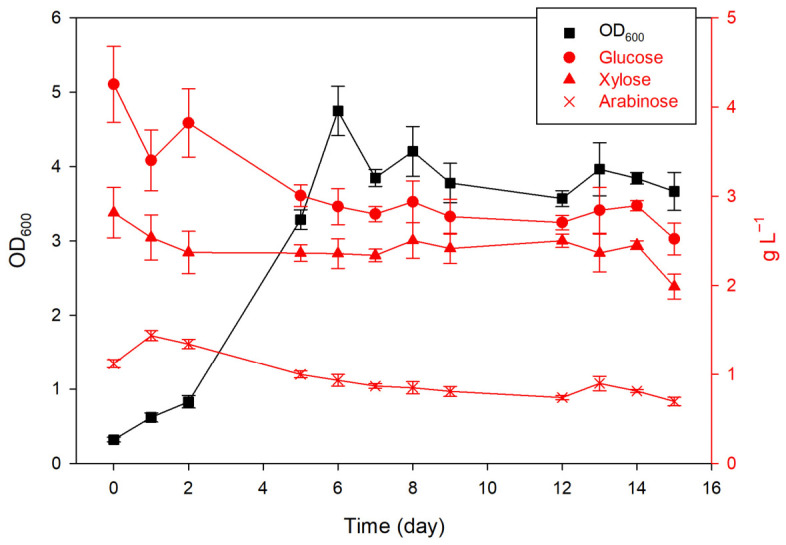
Profiles of optical density (OD_600_; black line), glucose, xylose, and arabinose concentrations (red lines) in the stirred-tank photobioreactor during the cultivation of *D. subspicatus*.

To illustrate the scalability of ryegrass enzymatic hydrolysate for algae cultivation, *D. subspicatus* was cultivated in a 1 L stirred-tank photobioreactor. BBM was supplemented with 25% alkaline-pretreated ryegrass enzymatic hydrolysate since it showed promising results in Erlenmeyer flasks. Therefore, the BBM medium contained around 5.79 g glucose, 2.80 g xylose, 1.74 g arabinose, and 1.24 g acetic acid, for a total of around 10 g L^−1^ of sugars. Results of optical density at 600 nm (Figure 4) showed exponential growth between day 2 and day 6, followed by a plateau. Microalgae cultivated in batch systems typically undergo several growth phases, including lag, exponential (lasting approximately 5–15 days), linear, declining, stationary, and death phases. The exponential phase is characterized by rapid cell division and biomass accumulation, while the stationary phase is associated with nutrient limitation, reduced protein synthesis, and the accumulation of storage compounds such as lipids and carbohydrates [52]. Dry matter concentration of the biomass and biomass productivity (Table 10) were 2.01 ± 0.04 g L^−1^ and 0.13 ± 0.01 g L^−1^ day^−1^, respectively, lower than in flasks on comparable medium. UPLC analysis of sugar consumption (Figure 4) and the calculated carbon source utilization of glucose, xylose, and arabinose (∆S, Table 10) during the cultivation of *D. subspicatus* in a stirred-tank photobioreactor showed that only around 30–40% of sugars were consumed. Meanwhile, almost complete consumption was recorded in flasks on a comparable medium. This reduced biomass growth and incomplete sugar consumption are likely due to inadequate gas–liquid mass transfer and elevated hydrodynamic shear stress induced by impeller mixing in the stirred-tank photobioreactor [53]. In flasks, gentle orbital shaking promotes better oxygen diffusion and nutrient access at the liquid surface. In contrast, intense bioreactor agitation can damage cell walls, disrupt metabolic uptake of sugars, and inhibit cell division in shear-sensitive microagae. Furthermore, the scale-up of photobioreactors introduces additional limitations related to heterogeneous light distribution and self-shading effects, which are recognized as key factors governing microalgal process performance [54]. Therefore, further optimization of mixing regimes, aeration rates, or impeller design would be undertaken to enhance substrate utilization and biomass productivity while minimizing shear stress. In addition, future studies should optimize key process parameters, such as the C/N ratio and substrate concentration, and improve light distribution within the photobioreactor to further enhance overall process efficiency.

#### 2.4.2. Fatty Acid Composition of *D. subspicatus*

Lipid content in *D. subspicatus* biomass cultivated in the stirred-tank photobioreactor was 22.05 ± 1.09%, which is much higher than that in comparable cultures in flasks (Table 11). However, due to the lower content of biomass, lipid productivity (Pr_L_) was two times lower than in flasks. Higher lipid content in photobioreactor cultures often results from nutrient limitation (e.g., incomplete sugar utilization, acting as a carbon/nitrogen stress) and environmental stressors such as shear or pH shifts, which trigger triacylglycerol accumulation as a protective mechanism [55,56].

While in flasks, the lipid profile was almost equally distributed among SFA, MUFA, and PUFA; in the photobioreactor, it was more shifted toward SFA (44.35 ± 2.49) and less toward PUFA (17.92 ± 1.46). The FAME profile mainly comprised C18:1 (35.48%), C16:0 (24.71%), C18:0 (13.96%) and C18:2 (10.54%) with minor amounts of C18:3 and C22:6. The shift toward higher SFA and lower PUFA observed in the photobioreactor is likely due to stress conditions (e.g., shear forces from mixing, light intensity, pH changes), leading cells to increase SFA/MUFA for enhanced membrane rigidity and energy storage while reducing peroxidation-prone PUFAs [57].

Scale-up cultivation of green microalgae on lignocellulosic biomass has been demonstrated in bioreactor systems. Arora and Philippidis (2021) cultivated *Chlorella vulgaris* on sweet sorghum bagasse in a 2 L bioreactor, achieving 6.9 g L^−1^ dry cell weight and 35% lipids [13], while Chen et al. (2015) used cassava bagasse for *Chlorella protothecoides* in 5 L and 50 L stirred bioreactors, reaching 2.8 g L^−1^ dry cell weight and 34.4% lipids [58]. These higher values than those for *D. subspicatus* (2.01 g L^−1^, 22.05%) likely reflect species-specific differences in substrate utilization and stress tolerance. This comparison also shows that *D. subspicatus* has the potential to be considered as a lipid producer at an industrial scale. However, further optimization of the bioprocess in the photobioreactor is needed.

## 3. Materials and Methods

### 3.1. Feedstock and Working Microorganism

In this study, the microalgae *Desmodesmus subspicatus* (CCAP 276/20), obtained from the Culture Collection of Algae and Protozoa (CCAP) (Dunbeg, Scotland, UK), was used as the working microorganism. Unless stated otherwise, all chemicals used in this research were sourced from Sigma-Aldrich (St. Louis, MO, USA).

The lignocellulosic feedstock used in this study was ryegrass (dry matter content 95.74 ± 1.26%) collected from the northwest part of Croatia (the vicinity of the town of Ludbreg). It was milled using a hammer mill and sieved through a 5 mm mesh (NA45; Megametal d.o.o., Kotoriba, Croatia). Milled ryegrass (500 g) was subjected to thermal pretreatment in a high-pressure reactor as described by Marđetko et al. [16]. Alkaline pretreatment was conducted with 10 L of 1% (w vol^−1^) NaOH solution at 180 °C for 10 min, while acid pretreatment was performed under identical conditions using 0.5% (w vol^−1^) H_2_SO_4_. The composition of ryegrass, alkaline-pretreated, and acid-pretreated ryegrass is shown in Table 12. After pretreatment, the solid fraction was separated, dried, and used for enzymatic hydrolysis.

Enzymatic hydrolysis was carried out using the commercially available enzyme mixtures, Viscozyme L and Cellulase from *Trichoderma reesei*. The reaction mixture contained 12 g of either alkaline- or acid-pretreated ryegrass in 300 mL of 50 mM sodium acetate buffer (pH 5), with the addition of 5% (*v*/*v*) Viscozyme and 2% (*v*/*v*) cellulase blend. Hydrolysis was performed at 40 °C on a magnetic stirrer for 48 h.

### 3.2. Nutrient Medium for Microalgae Cultivation

This study investigated the growth of *D. subspicatus* under varying carbon and nitrogen conditions, as well as in the presence of ryegrass-derived enzymatic hydrolysates, following the experimental design presented in Table 13.

Liquid Bold’s Basal Medium (BBM) was used for all cultivations, and its composition is presented in Table A1. To investigate the effect of carbon source and concentration, BBM was supplemented with glucose at 5, 10, 25, and 50 g L^−1^, or with xylose and arabinose at 5, 10, and 25 g L^−1^. As a control, BBM medium without an added carbon source was used.

To examine and optimize the influence of different nitrogen sources and different C/N ratios, BBM medium containing 10 g L^−1^ of glucose was supplemented with either NaNO_3_ or yeast extract to achieve C/N ratios of 10:1, 30:1 or 50:1. The amounts of nitrogen sources were calculated based on their nitrogen content, assuming 10% nitrogen (*w*/*w*) in yeast extract (Y1625, Sigma-Aldrich, St. Louis, MO, USA) according to the manufacturer. They were adjusted to provide equivalent nitrogen levels. BBM with 10 g L^−1^ glucose but without an added nitrogen source was used as the control.

To assess and optimize the usage of hydrolysates, BBM was supplemented with 1, 5, 10, 25, or 50% hydrolysate obtained after alkaline or acid pretreatment (Section 3.1). BBM medium without the hydrolysate was used as the control.

### 3.3. Optimization of Microalgae Growth on Different Carbon and Nitrogen Sources

Cultivations under mixotrophic conditions were carried out in 500 mL Erlenmeyer flasks containing 200 mL of sterile BBM nutrient medium supplemented with the appropriate carbon source, nitrogen source, or ryegrass enzymatic hydrolysate (Table 1). Each flask was inoculated with 20 mL (10% *v*/*v*) of the pre-cultivated *D. subspicatus* inoculum. The cultivations were performed on an orbital shaker (Certomat RM 71, B. Braun Biotech International, Hessen, Germany) at 180 rpm, 28 °C, without aeration, under continuous warm white illumination (light intensity not determined). All experiments were performed in the same illumination conditions. The cultivation period lasted from 15 to 29 days, during which samples for further analysis were withdrawn at intervals of 2–3 days. All experiments were performed at least in duplicate, and the results are expressed as mean values of the measured data.

### 3.4. Cultivation of D. subspicatus in a Stirred-Tank Photobioreactor

Cultivation of *D. subspicatus* was performed under mixotrophic conditions in a stirred-tank photobioreactor (Biostat MD, B. Braun Biotech International, Germany) equipped with a white-light source. The medium for microalgae cultivation was BBM supplemented with 25% alkaline-pretreated ryegrass enzymatic hydrolysate. The photobioreactor was filled with medium and sterilized in an autoclave at 121 °C for 15 min. After cooling, the photobioreactor was inoculated with a 10% *v*/*v* suspension of *D. subspicatus* previously cultivated on a rotary shaker (see Section 2.3). The cultivation was performed at 180 rpm, 28 °C, pH around 6.5, with aeration, under continuous warm white illumination (light intensity was not determined). During the 15-day cultivation period, samples were collected at 2–3-day intervals for monitoring bioprocess performance. After cultivation, biomass was separated from the medium by centrifugation.

### 3.5. Analytical Methods

#### 3.5.1. Measurement of Optical Density

The growth of *D. subspicatus* was monitored throughout cultivation by periodically sampling the culture medium and measuring its optical density (OD) at 600 nm using a UV-Vis spectrophotometer (Cary 100, UV-Vis, Agilent Technologies, Santa Clara, CA, USA). Measurements were performed in 2 mL glass cuvettes. To ensure the OD values remained within the linear range of 0–1, samples were diluted with distilled water as needed.

#### 3.5.2. Gravimetric Determination of Dry Matter Concentration

For microalgae cultivated with hydrolysate supplementation, dry matter concentration was also determined gravimetrically. A defined volume of culture medium was transferred into pre-dried, pre-weighed plastic cuvettes. The cuvettes containing the samples were centrifuged at 8000 rpm for 10 min to separate the biomass. The supernatant was decanted, and the remaining biomass pellet was resuspended in distilled water to remove residual medium components and salts. After another round of centrifugation under the same conditions, the supernatant was again decanted, and the biomass pellets were dried at 100 °C until a constant weight was achieved. The dried cuvettes were cooled in a desiccator and subsequently weighed. The dry matter concentration of the microalgal biomass was calculated using Equation (1):

(1) $$X=\;\frac{m_{2}-m_{1}}{V_{m}}$$

where the following applies:

X—the dry matter concentration of the biomass (g L^−1^),

m_1_—the mass of the empty dried cuvette (g),

m_2_—the mass of the cuvette with the dried biomass (g),

V_m_—the volume of the medium taken for analysis (L).

#### 3.5.3. UPLC Analysis

The composition of carbohydrates, alcohols, and organic acids during cultivation was quantified using ultra-performance liquid chromatography (UPLC) (Agilent Technologies 1290 Infinity II, Santa Clara, CA, USA). The system was equipped with a pre-column, Rezex ROA-Organic Acid H^+^ analytical column (150 × 7.8 mm, Phenomenex, Torrance, CA, USA), a refractive index detector (G7162A 1260 RID), a pump (G7104A 1290 Flexible Pump), a column oven, an autosampler (G7129B 1290 Vialsampler), and chromatography data software (OpenLAB CDS).

Culture samples collected throughout the cultivation were centrifuged (SL 8R Thermoscientific, Waltham, MA, USA) to obtain the supernatant, which was then filtered into analytical vials using a nylon syringe filter (CHROMAFIL Xtra PA-20/25, Düren, Germany) with a 0.2 μm pore size. The prepared samples were analyzed following the method described by Marđetko et al. [59]. A 10 μL aliquot of each sample was injected into the UPLC system. Isocratic elution was carried out using 0.0025 M H_2_SO_4_ as the mobile phase at a flow rate of 0.6 mL min^−1^. The column oven temperature was maintained at 60 °C, and the RID temperature at 40 °C.

#### 3.5.4. Determination of Fatty Acid Composition in Microalgal Biomass

Fatty acids were transesterified following the National Renewable Energy Laboratory (NREL) [60] protocol for total lipid analysis. Dried and milled biomass (5–10 mg) was weighed into glass vials and spiked with 20 μL of methyl tridecanoate (C13:0ME; 10 mg mL^−1^ in hexane) as the internal standard. Then, 200 µL of chloroform/methanol solution (2:1, *v*/*v*) and 300 µL of methanolic HCl (0.6 M) were added. The vials were sealed, vortexed (Technica ET-1111, Slovenia), and incubated in a water bath (WSB-18, Witeg, Wertheim, Germany) at 85 °C for 1 h. After cooling to room temperature, 1 mL of hexane was added, and the samples were vortexed and left for 1 h at room temperature to allow phase separation. The upper hexane layer (400 µL), containing the fatty acid methyl esters (FAMEs), was transferred to clean vials along with 10 μL of pentadecane (1 mg mL^−1^ in hexane) as an additional standard.

The FAME samples were analyzed using a gas chromatography system (GC 2010 Plus AF, Shimadzu, Kyoto, Japan) equipped with an autosampler (AOC-20s), an injector (AOC-20i), and a thermostatted capillary column (Zebron ZBFAME, Phenomenex, Torrance, CA, USA). Method parameters are presented in Table 14. Detection was performed using a flame ionization detector (FID-2010 Plus), while data acquisition and processing were carried out using the GC Solutions v2.32 software (Shimadzu, Kyoto, Japan).

Identification of fatty acids in the samples was achieved by comparing the retention times of the corresponding methyl esters with those of a 37-component FAME standard mixture (C4–C24) analyzed under the same chromatographic conditions. The total fatty acid content was calculated according to Equation (2):

(2) $${totalFAME}_{C13norm}=\sum_{C4-C24\frac{{FAME}_{Ci}}{{FAME}_{C13}}\times c_{addedFAMEC13}}$$

Total fatty acids were expressed as a percentage of the dry biomass used for transesterification, as shown in Equation (3):

(3) $$\%\mathrm{total}\mathrm{FAME}=\frac{{\mathrm{total}\mathrm{FAME}}_{\;C13norm}}{m}\;\times 100$$

where the following applies:

FAME_Ci_—the amount of the measured FAME,

FAME_C13_—the amount of the measured C13 standard,

c_addedFAMEC13_—the amount of the C13 internal standard added at the beginning of the transesterification process,

m—mass of dry biomass (g).

#### 3.5.5. Determination of Protein Content in Biomass

The protein content of the microalgal biomass was quantified by the Lowry method. Biomass samples (5 mg) were resuspended in 1 mL of distilled water, and a 20 µL aliquot was further diluted with 0.5 mL of water in a glass tube. Subsequently, 0.5 mL of 1 M NaOH was added, the tubes were sealed, and the samples were heated in a water bath at 85 °C for 5 min, followed by cooling to room temperature. Then, 2.5 mL of a freshly prepared reagent mixture was added, prepared by mixing 5% Na_2_CO_3_ with 0.5% CuSO_4_ in 1% potassium–sodium tartrate in a 50:1 ratio. After 10 min, 0.5 mL of diluted Folin–Ciocalteu reagent (1:1 *v*/*v* in water) was added, and the tubes were incubated in the dark at room temperature for 40 min. Absorbance was measured at 550 nm using a UV/VIS spectrophotometer (Cary 100, UV-VIS, Agilent Technologies, Santa Clara, CA, USA). A blank was prepared using distilled water instead of the biomass sample. Protein concentration was determined using a calibration curve prepared from bovine serum albumin standards (0–100 mg L^−1^). The protein content in the biomass was calculated using Equation (4):

(4) $$W_{p}=\frac{m_{p}}{m_{X}}\times 100\%$$

where the following applies:

W_p_—protein content in biomass (%),

m_p_—protein mass (mg),

m_X_—biomass mass used (mg).

#### 3.5.6. Determination of Carbohydrate Content in Biomass

Carbohydrate content in the microalgal biomass was determined using the method developed by the NREL [61]. Briefly, 10–25 mg of dried and milled biomass was treated with 250 µL of 72% H_2_SO_4_ and incubated at 30 °C for 1 h with occasional mixing. Afterwards, 7 mL of deionized water was added, the vials were sealed with rubber stoppers, reinforced with aluminum caps, and autoclaved at 121 °C for 1 h. After cooling to room temperature, a 3 mL aliquot of the hydrolysate was neutralized to pH 5 with CaCl_2_, filtered through a 0.22 µm syringe filter, and analyzed by UPLC as described in Section 3.5.3. The carbohydrate content of the biomass was calculated using Equation (5).

(5) $$W_{C}=\frac{m_{C}}{m_{X}}$$

where the following applies:

W_C_—carbohydrate content in biomass (%),

m_C_—mass of carbohydrates (mg),

m_X_—mass of biomass (mg).

#### 3.5.7. Determination of Chlorophyll Content in Biomass

Chlorophyll-a (Chl_a_) and chlorophyll-b (Chl_b_) were extracted from 10 mg of dried biomass using 2 mL of acetone and 2 g of glass beads (B. Braun Biotech International GmbH, Germany). Test tubes were wrapped in aluminum foil to protect photosensitive pigments from light. The sample was vortexed for 30 s, cooled on ice between vortexing steps, and subsequently centrifuged at 5000 rpm for 10 min. The supernatant was transferred into a clean test tube wrapped in aluminum foil, and the pellet was re-extracted until complete decolorization of the biomass was achieved. Supernatants from each extraction step were pooled. Chl_a_ and Chl_b_ were determined spectrophotometrically according to Equations (6)–(8) [62]:

(6) $${Chl}_{a}=11.24A_{662}-2.04A_{645}$$

(7) $${Chl}_{b}=22.13A_{645}-4.19A_{662}$$

(8) $${Chl}_{total}={Chl}_{a}+{Chl}_{b}$$

where the following applies:

Chl_a_—chlorophyll-a content (μg mL^−1^),

Chl_b_—chlorophyll-b content (μg mL^−1^),

Chl_total_—sum of chlorophyll-a and chlorophyll-b (μg mL^−1^),

A_645_—absorbance at 645 nm,

A_662_—absorbance at 662 nm.

#### 3.5.8. Determination of Total Polyphenol Content in Biomass

Polyphenols were extracted by adding 0.6 mL of methanol to 60 mg of dry biomass in test tubes, then sonifying for 1 h in an ultrasonic bath. The supernatant was collected, and the pellet was re-extracted with a fresh 0.6 mL of methanol under the same conditions. The resulting supernatants were pooled. Total polyphenol content (TPC) was determined spectrophotometrically using the Folin–Ciocalteu method, as previously described [63]. Briefly, 100 µL of each extract was combined with 200 µL of Folin–Ciocalteu reagent and 2 mL of distilled water. After 3 min, 1 mL of saturated Na_2_CO_3_ solution was added, and the mixture was briefly vortexed. Samples were then incubated in a 50 °C water bath for 25 min, after which absorbance was measured at 765 nm. TPC was calculated from a gallic acid calibration curve and expressed as mg gallic acid equivalents (GAE) per g of dry biomass.

### 3.6. Determination of Process Efficiency Parameters

The efficiency of the *D. subspicatus* cultivation was evaluated using the following parameters [64]:

Carbon Source Consumption (ΔS [%])

(9) $$\Delta S\left[\%\right]=\left(1-\frac{S}{S_{0}}\right)*100\%$$

S_0_—substrate concentration at the beginning of cultivation (g L^−1^)

S—substrate concentration at the end of cultivation (g L^−1^)

Biomass Productivity (Pr_X_ [g L^−1^ day^−1^])

(10) $${Pr}_{X}=\frac{X}{t}$$

X—dry matter concentration of the biomass (g L^−1^)

t—cultivation time (day)

Lipid Productivity (Pr_L_ [g L^−1^ day^−1^])

(11) $${Pr}_{L}=\frac{X\times W_{L}}{t}$$

X—dry matter concentration of the biomass (g L^−1^)

W_L_—lipid fraction in the biomass at time t (%)

t—cultivation time (day)

### 3.7. Statistical Analysis

Standard deviations of the experimental data were calculated using Statistica 12.0 (StatSoft, Tulsa, OK, USA). All experiments were performed at least in duplicate, and results are presented as mean values ± standard deviations. The residuals of the data were assessed for normality and homogeneity of variance. Differences among treatments were evaluated using one-way analysis of variance (ANOVA) followed by Tukey’s HSD post hoc test. A significance level of *p* ≤ 0.05 was applied for all statistical tests.

## 4. Conclusions

This study demonstrates that *Desmodesmus subspicatus* has the potential to be used as an industrial strain for lipid production. It can be effectively cultivated under mixotrophic conditions using either simple sugars or ryegrass-derived lignocellulosic hydrolysates. The carbon source, nitrogen source, C/N ratio, ryegrass pretreatment method, and ryegrass hydrolysate concentration have significant and differentiating impacts on biomass growth, lipid accumulation, and overall biochemical composition. Glucose and arabinose supported strong biomass accumulation, whereas xylose led to weak growth accompanied by the loss of pigments. Glucose significantly increased cellular lipid content (≥24%, up to 46%), and the fatty acid profile was dominated by C16 and C18 fatty acids (palmitic, oleic, linoleic, α-linolenic), which are important for biodiesel quality. Nitrogen limitation at higher C/N ratios promoted lipid accumulation, sodium nitrate outperformed yeast extract in total lipid content, while yeast extract shifted profiles toward PUFA. When used as an alternative carbon source, the ryegrass enzymatic hydrolysate obtained after alkaline pretreatment supported strong, dose-dependent biomass growth, reaching approximately 12 g L^−1^ at 50%, with peak lipid productivity. Meanwhile, the acid-pretreated hydrolysate inhibited growth at 50%. Hydrolysate addition generally decreased protein and chlorophyll contents but increased carbohydrate fractions, indicating a metabolic shift toward storage compounds under mixotrophy. Scale-up in a 1 L stirred-tank photobioreactor using 25% ryegrass hydrolysate yielded lower biomass but higher lipid content, with a fatty acid profile shifted toward SFA, underscoring the need for bioprocess optimization to improve overall performance. Our findings support the use of ryegrass as an alternative carbon source and identify parameters that steer growth and fuel-relevant lipid profiles. They also provide guidance for designing economically viable lignocellulosic microalgal processes and indicate the need for scale-up studies with different cultivation manners (e.g., fed batch, repeated batch, repeated fed batch, or continuous) to maximize bioprocess efficiency and biofuel properties.

## Figures and Tables

**Table 1:** Carbon source consumption (∆S) by *D. subspicatus* cultivated on different carbon sources.

	G5	G10	G25	G50	X5	X10	X25	A5	A10	A25
∆S (%) *p* < 0.001 *	47.29 ± 1.42 ^b^	20.93 ± 0.96 ^c^	17.79 ± 0.78 ^c^	6.34 ± 0.33 ^d^	93.47 ± 2.68 ^a^	23.70 ± 1.24 ^c^	20.89 ± 1.31 ^c^	97.64 ± 2.54 ^a^	97.02 ± 2.24 ^a^	59.46 ± 1.49 ^b^

* *p* ≤ 0.05. Means with different letters within a row are significantly different (*p* ≤ 0.05).

**Table 2:** Fatty acid profile of *D. subspicatus* cultivated on media with different carbon sources.

	w (%)							
	BBM	G5	G10	G25	G50	A5	A10	A25
C11:0	0.26 ± 0.05	0.09 ± 0.01	0.26 ± 0.03	0.19 ± 0.04	0.15 ± 0.03	0.42 ± 0.07	0.35 ± 0.04	0.40 ± 0.04
C14:0	0.05 ± 0.01	0.11 ± 0.01	0.03 ± 0.01	0.07 ± 0.01	0.13 ± 0.02	-	-	-
C14:1 cis 9	0.53 ± 0.02	0.13 ± 0.02	0.01 ± 0.01	-	-	-	-	-
C15:0	-	-	1.14 ± 0.23	0.74 ± 0.04	-	-	0.02 ± 0.01	-
C15:1 cis 10	0.32 ± 0.03	0.07 ± 0.01	0.06 ± 0.01	-	-	-	-	-
C16:0	25.59 ± 0.56	30.24 ± 0.52	32.40 ± 0.67	31.97 ± 0.86	30.41 ± 0.69	16.62 ± 0.49	32.60 ± 0.42	32.63 ± 0.53
C16:1 cis 9	4.67 ± 0.22	0.89 ± 0.08	2.55 ± 0.26	1.43 ± 0.14	2.00 ± 0.21	-	0.32 ± 0.02	0.38 ± 0.02
C17:0	2.62 ± 0.14	3.55 ± 0.17	6.10 ± 0.47	5.03 ± 0.27	6.61 ± 0.51	-	-	-
C17:1 cis 10	0.24 ± 0.01	0.09 ± 0.01	0.24 ± 0.03	0.08 ± 0.01	0.13 ± 0.01	-	-	-
C18:0	1.17 ± 0.14	2.09 ± 0.25	1.39 ± 0.15	1.68 ± 0.24	2.08 ± 0.11	8.48 ± 0.21	23.57 ± 0.76	26.71 ± 0.31
C18:1 trans 9	0.70 ± 0.04	0.11 ± 0.01	-	-	0.06 ± 0.01	-	-	-
C18:1 cis 9	24.06 ± 0.39	23.68 ± 0.35	18.11 ± 0.56	22.81 ± 0.52	23.74 ± 0.55	21.09 ± 0.48	32.44 ± 0.88	26.68 ± 0.46
C18:2 trans 9,12	-	-	0.53 ± 0.03	0.26 ± 0.04	0.23 ± 0.05	-	-	-
C18:2 cis 9, 12	11.70 ± 0.54	21.02 ± 0.63	18.74 ± 0.68	20.11 ± 0.63	18.58 ± 0.69	42.01 ± 0.95	-	-
C18:3 cis 6, 9, 12	2.67 ± 0.12	2.31 ± 0.12	2.61 ± 0.29	2.37 ± 0.27	1.97 ± 0.15	-	-	-
C18:3 cis 9,12,15	21.69 ± 0.78	14.24 ± 0.44	12.39 ± 0.24	11.04 ± 0.24	8.20 ± 0.38	5.37 ± 0.36	4.83 ± 0.26	3.61 ± 0.34
C20:0	-	0.38 ± 0.02	1.06 ± 0.54	0.87 ± 0.08	0.90 ± 0.12	1.32 ± 0.14	1.12 ± 0.25	1.13 ± 0.14
C20:1 cis 11	0.39 ± 0.02	0.49 ± 0.02	1.41 ± 0.24	0.90 ± 0.08	0.54 ± 0.06	0.50 ± 0.06	-	-
C20:2 cis 11, 14	0.36 ± 0.02	0.04 ± 0.01	0.38 ± 0.03	-	0.12 ± 0.02	0.83 ± 0.21	0.50 ± 0.03	-
C20:3 cis 11, 14, 17	0.13 ± 0.01	0.03 ± 0.01	-	-	-	-	-	-
C22:0	0.15 ± 0.01	0.04 ± 0.01	-	-	-	-	0.73 ± 0.06	-
C22:2 cis 13, 16	0.15 ± 0.01	-	-	-	0.64 ± 0.08	-	-	-
C23:0	0.30 ± 0.03	0.12 ± 0.01	-	-	-	1.10 ± 0.12	-	-
C24:0	1.29 ± 0.24	0.15 ± 0.03	-	-	1.08 ± 0.24	0.89 ± 0.06	1.24 ± 0.17	1.02 ± 0.08
C24:1 cis 15	0.44 ± 0.03	-	-	-	0.83 ± 0.09	-	-	-
C22:6 cis 4, 7, 10, 13, 16, 19	0.19 ± 0.02	0.11 ± 0.01	0.58 ± 0.04	0.45 ± 0.02	0.95 ± 0.12	1.37 ± 0.14	2.28 ± 0.21	7.44 ± 0.14
Σ SFA *p* < 0.001 *	31.53 ± 0.15 ^d^	36.78 ± 0.11 ^c^	42.38 ± 0.30 ^b^	40.56 ± 0.22 ^b^	41.63 ± 0.25 ^b^	28.83 ± 0.18 ^e^	59.63 ± 0.24 ^a^	61.89 ± 0.26 ^a^
Σ MUFA *p* < 0.001 *	31.47 ± 0.10 ^a^	25.47 ± 0.07 ^c^	22.38 ± 0.19 ^d^	25.22 ± 0.19 ^c^	27.48 ± 0.16 ^b^	21.59 ± 0.27 ^d^	32.76 ± 0.45 ^a^	27.06 ± 0.24 ^b^
Σ PUFA *p* < 0.001 *	37.00 ± 0.21 ^b^	37.74 ± 0.20 ^b^	35.24 ± 0.22 ^c^	34.22 ± 0.24 ^c^	30.89 ± 0.21 ^d^	49.58 ± 0.27 ^a^	7.61 ± 0.26 ^f^	11.05 ± 0.23 ^e^
W_L_ (%) *p* < 0.001 *	6.45 ± 0.15 ^e^	45.60 ± 0.13 ^a^	24.79 ± 0.24 ^d^	37.80 ± 0.22 ^b^	44.29 ± 0.21 ^a^	13.31 ± 0.27 ^f^	13.50 ± 0.26 ^f^	19.04 ± 0.23 ^e^

W_L_ (%)—lipid content in biomass. * *p* ≤ 0.05. Means with different letters within a row are significantly different (*p* ≤ 0.05).

**Table 3:** Carbon source consumption (∆S) by *D. subspicatus* cultivated on different nitrogen sources and carbon/nitrogen ratio.

	CONTROL	NO10	NO30	NO50	YE10	YE30	YE50
∆S (%) *p* < 0.001 *	19.44 ± 1.26 ^b^	99.51 ± 2.67 ^a^	99.26 ± 2.96 ^a^	98.73 ± 2.88 ^a^	99.85 ± 3.16 ^a^	99.50 ± 2.98 ^a^	99.27 ± 2.79 ^a^

* *p* ≤ 0.05. Means with different letters are significantly different (*p* ≤ 0.05).

**Table 4:** Fatty acid profile of *D. subspicatus* cultivated on media with different nitrogen sources and carbon to nitrogen ratio.

	w (%)						
	CONTROL	NO10	NO30	NO50	YE10	YE30	YE50
C8:0	-	9.91 ± 0.22	1.98 ± 0.11	-	-	-	1.19 ± 0.19
C10:0	-	3.19 ± 0.17	0.63 ± 0.18	-	2.76 ± 0.18	-	0.38 ± 0.03
C11:0	0.39 ± 0.22	1.31 ± 0.17	0.34 ± 0.03	0.51 ± 0.19	1.28 ± 0.19	0.20 ± 0.03	0.14 ± 0.02
C15:0	-	-	-	0.27 ± 0.14	-	-	-
C16:0	21.46 ± 0.52	19.33 ± 0.46	16.22 ± 0.41	18.03 ± 0.44	13.22 ± 0.46	11.14 ± 0.62	15.33 ± 0.55
C16:1 cis 9	6.18 ± 0.31	-	0.49 ± 0.23	0.25 ± 0.04	-	0.46 ± 0.04	0.45 ± 0.08
C17:0	6.30 ± 0.36	0.37 ± 0.09	0.03 ± 0.01	-	2.40 ± 0.18	-	0.60 ± 0.05
C18:0	-	1.77 ± 0.19	4.32 ± 0.22	7.44 ± 0.23	1.22 ± 0.17	2.99 ± 0.19	4.08 ± 0.21
C18:1 cis 9	5.62 ± 0.24	26.93 ± 0.45	24.04 ± 0.42	22.22 ± 0.48	19.46 ± 0.46	25.93 ± 0.45	24.65 ± 0.46
C18:2 trans 9,12	-	-	-	0.74 ± 0.08	-	-	-
C18:2 cis 9, 12	19.64 ± 0.26	32.89 ± 0.71	45.81 ± 0.67	27.59 ± 0.55	41.88 ± 0.89	51.92 ± 0.78	47.11 ± 0.69
C18:3 cis 6, 9, 12	3.78 ± 0.11	-	0.22 ± 0.02	1.56 ± 0.17	-	0.13 ± 0.02	0.09 ± 0.01
C18:3 cis 9,12,15	28.57 ± 0.29	3.64 ± 0.25	4.67 ± 0.33	8.62 ± 0.46	0.53 ± 0.08	5.11 ± 0.34	5.43 ± 0.24
C20:0	-	-	0.36 ± 0.03	3.11 ± 0.21	-	0.01 ± 0.01	0.30 ± 0.03
C20:1 cis 11	7.21 ± 0.15	-	-	-	0.14 ± 0.09	-	0.04 ± 0.01
C20:2 cis 11, 14	-	0.18 ± 0.03	-	-	0.03 ± 0.01	-	-
C20:3 cis 11, 14, 17	-	5.24 ± 0.24	0.12 ± 0.02	-	5.31 ± 0.23	0.11 ± 0.01	0.03 ± 0.01
C22:0	-	-	0.17 ± 0.02	-	0.23 ± 0.21	0.18 ± 0.03	0.27 ± 0.04
C22:2 cis 13, 16	-	0.14 ± 0.03	0.40 ± 0.03	-	0.16 ± 0.02	0.20 ± 0.03	0.43 ± 0.04
C23:0	0.32 ± 0.04	5.43 ± 0.29	1.89 ± 0.19	1.31 ± 0.17	5.40 ± 0.24	0.85 ± 0.26	0.11 ± 0.02
C24:0	0.33 ± 0.05	2.01 ± 0.17	0.65 ± 0.06	0.97 ± 0.14	3.58 ± 0.18	0.62 ± 0.29	0.60 ± 0.32
C24:1 cis 15	-	0.62 ± 0.25	0.09 ± 0.01	6.07 ± 0.36	0.40 ± 0.08	0.10 ± 0.01	0.09 ± 0.01
C22:6 cis 4, 7, 10, 13, 16, 19	0.19 ± 0.02	0.14 ± 0.16	0.11 ± 0.02	1.31 ± 0.18	4.76 ± 0.16	0.03 ± 0.01	0.08 ± 0.01
Σ SFA *p* < 0.001 *	28.81 ± 0.24 ^c^	38.30 ± 0.22 ^a^	25.93 ± 0.13 ^d^	31.64 ± 0.22 ^b^	29.28 ± 0.23 ^c^	15.99 ± 0.20 ^f^	22.68 ± 0.15 ^e^
Σ MUFA *p* < 0.001 *	19.01 ± 0.23 ^d^	24.37 ± 0.35 ^b^	24.01 ± 0.22 ^b^	28.55 ± 0.22 ^a^	19.46 ± 0.21 ^d^	26.50 ± 0.17 ^a^	24.87 ± 0.14 ^b^
Σ PUFA *p* < 0.001 *	52.18 ± 0.17 ^b^	37.34 ± 0.24 ^d^	50.06 ± 0.18 ^c^	39.81 ± 0.29 ^d^	51.26 ± 0.23 ^b^	57.51 ± 0.20 ^a^	52.45 ± 0.17 ^b^
W_L_ (%) *p* < 0.001 *	5.54 ± 0.21 ^d^	1.59 ± 0.24 ^f^	7.92 ± 0.17 ^c^	17.66 ± 0.26 ^a^	1.28 ± 0.23 ^f^	11.39 ± 0.20 ^b^	13.84 ± 0.15 ^b^

W_L_ (%)—lipid content in biomass. * *p* ≤ 0.05. Means with different letters within a row are significantly different (*p* ≤ 0.05).

**Table 5:** Composition of perennial ryegrass (*Lolium perenne*) enzymatic hydrolysate obtained after alkaline and acid pretreatment.

g L^−1^	Alkaline Pretreatment	Acid Pretreatment
Glucose	23.15 ± 1.24	18.39 ± 1.07
Xylose	11.21 ± 0.97	7.11 ± 0.65
Arabinose	6.94 ± 0.47	7.14 ± 0.78
Acetic acid	4.95 ± 0.33	5.53 ± 0.69

**Table 6:** Dry matter concentration of the biomass (g L^−1^) and biomass productivity (g L^−1^ day^−1^) at the end of the cultivation period (day 15).

	BBM	AL1	AL5	AL10	AL25	AL50	AC1	AC5	AC10	AC25	AC50
X (g L^−1^) *p* < 0.001 *	0.41 ± 0.09 ^h^	1.05 ± 0.04 ^f^	1.73 ± 0.04 ^f^	2.74 ± 0.08 ^d^	6.32 ± 0.09 ^b^	12.03 ± 0.14 ^a^	0.73 ± 0.17 ^g^	1.10 ± 0.21 ^f^	2.05 ± 0.26 ^d^	4.40 ± 0.26 ^c^	0.35 ± 0.09 ^h^
Pr_X_ (g L^−1^ day^−1^) *p* < 0.001 *	0.03 ± 0.01 ^h^	0.07 ± 0.00 ^f^	0.12 ± 0.00 ^e^	0.18 ± 0.01 ^d^	0.42 ± 0.01 ^b^	0.80 ± 0.02 ^a^	0.05 ± 0.02 ^g^	0.07 ± 0.02 ^f^	0.14 ± 0.02 ^d^	0.29 ± 0.02 ^c^	0.02 ± 0.03 ^h^

* *p* ≤ 0.05. Means with different letters within a row are significantly different (*p* ≤ 0.05).

**Table 7:** Carbon source consumption (∆S) by *D. subspicatus* cultivated on media with different ryegrass enzymatic hydrolysate content.

∆S (%)	AL1	AL5	AL10	AL25	AL50	AC1	AC5	AC10	AC25	AC50
Glucose *p* < 0.001 *	44.89 ± 0.81 ^d^	95.05 ± 2.88 ^b^	97.45 ± 1.99 ^a,b^	99.19 ± 2.12 ^a^	99.07 ± 2.14 ^a^	63.34 ± 0.86 ^c^	86.93 ± 0.65 ^b^	94.81 ± 2.25 ^b^	98.36 ± 1.45 ^a^	0.01 ± 0.01 ^e^
Xylose *p* < 0.001 *	97.05 ± 1.24 ^a,b^	99.91 ± 1.36 ^a^	99.88 ± 1.84 ^a^	99.84 ± 2.23 ^a^	95.96 ± 1.62 ^b^	99.87 ± 1.49 ^a^	90.51 ± 2.26 ^c^	99.73 ± 1.55 ^a^	99.02 ± 2.48 ^a^	1.61 ± 0.01 ^d^
Arabinose *p* < 0.001 *	49.32 ± 0.84 ^d^	98.29 ± 1.98 ^a,b^	99.44 ± 1.23 ^a^	95.60 ± 1.46 ^b^	91.17 ± 1.62 ^c^	86.64 ± 1.55 ^c^	99.53 ± 2.21 ^a^	99.78 ± 2.64 ^a^	87.32 ± 2.56 ^c^	1.75 ± 0.02 ^e^

* *p* ≤ 0.05. Means with different letters within a row are significantly different (*p* ≤ 0.05).

**Table 8:** Fatty acids profile and lipid productivity (Pr_L_) of *D. subspicatus* cultivated on media with different ryegrass enzymatic hydrolysate content.

	w (%)							
	AL1	AL5	AL10	AL25	AL50	AC5	AC10	AC25
C8:0	-	2.97 ± 0.13	2.84 ± 0.13	2.42 ± 0.11	1.02 ± 0.11	3.08 ± 0.12	3.98 ± 0.11	4.16 ± 0.09
C10:0	-	0.95 ± 0.11	0.93 ± 0.14	0.82 ± 0.10	0.04 ± 0.01	0.98 ± 0.11	1.29 ± 0.14	1.41 ± 0.04
C11:0	0.33 ± 0.04	0.35 ± 0.05	0.32 ± 0.02	0.36 ± 0.04	0.17 ± 0.02	0.36 ± 0.07	0.48 ± 0.06	0.57 ± 0.09
C16:0	26.94 ± 0.29	31.26 ± 0.35	30.92 ± 0.49	25.46 ± 0.24	23.72 ± 0.21	23.47 ± 0.27	29.05 ± 0.19	18.40 ± 0.16
C16:1 cis 9	2.94 ± 0.22	1.03 ± 0.14	0.29 ± 0.05	1.76 ± 0.14	1.63 ± 0.17	0.78 ± 0.12	0.40 ± 0.07	1.28 ± 0.09
C17:0	5.36 ± 0.14	4.77 ± 0.16	-	0.19 ± 0.03	0.04 ± 0.01	3.18 ± 0.09	0.04 ± 0.01	0.15 ± 0.03
C18:0	0.93 ± 0.15	2.38 ± 0.18	2.38 ± 0.19	2.73 ± 0.23	6.19 ± 0.19	1.40 ± 0.12	2.68 ± 0.19	3.02 ± 0.21
C18:1 cis 9	19.87 ± 0.19	24.63 ± 0.36	31.16 ± 0.42	32.96 ± 0.32	28.10 ± 0.25	16.54 ± 0.21	25.53 ± 0.18	31.39 ± 0.28
C18:2 cis 9, 12	24.41 ± 0.22	22.72 ± 0.29	30.26 ± 0.39	35.20 ± 0.36	37.96 ± 0.33	14.53 ± 0.19	28.15 ± 0.18	42.88 ± 0.41
C18:3 cis 6, 9, 12	1.45 ± 0.16	0.71 ± 0.09	0.55 ± 0.06	-	-	0.51 ± 0.06	10.04 ± 0.11	-
C18:3 cis 9,12,15	17.18 ± 0.19	10.19 ± 0.24	3.68 ± 0.19	0.43 ± 0.05	0.49 ± 0.08	10.92 ± 0.12	0.34 ± 0.03	0.93 ± 0.07
C20:0	-	0.43 ± 0.06	-	-	0.32 ± 0.02	0.33 ± 0.03	-	-
C20:1 cis 11	0.13 ± 0.01	0.19 ± 0.03	-	-	-	0.08 ± 0.01	-	-
C20:2 cis 11, 14	-	0.75 ± 0.06	-	0.01 ± 0.01	0.07 ± 0.01	0.71 ± 0.11	-	-
C20:3 cis 11, 14, 17	-	-	-	0.05 ± 0.01	-	-	0.21 ± 0.02	0.01 ± 0.01
C22:0	-	-	-	0.08 ± 0.01	0.32 ± 0.09	-	0.10 ± 0.01	0.18 ± 0.01
C22:2 cis 13, 16	0.09 ± 0.01	0.12 ± 0.01	0.04 ± 0.01	0.12 ± 0.01	0.10 ± 0.01	0.08 ± 0.01	0.20 ± 0.01	0.10 ± 0.01
C23:0	-	0.06 ± 0.01	0.09 ± 0.01	0.07 ± 0.01	0.14 ± 0.01	0.40 ± 0.06	0.80 ± 0.11	0.10 ± 0.01
C24:0	0.26 ± 0.02	0.25 ± 0.02	0.21 ± 0.02	0.45 ± 0.13	0.55 ± 0.11	0.40 ± 0.08	1.09 ± 0.16	0.04 ± 0.01
C24:1 cis 15	0.11 ± 0.01	-	0.11 ± 0.01	0.09 ± 0.01	0.12 ± 0.01	0.20 ± 0.02	0.74 ± 0.11	0.52 ± 0.08
C22:6 cis 4, 7, 10, 13, 16, 19	-	0.11 ± 0.01	-	0.04 ± 0.01	0.07 ± 0.01	0.11 ± 0.01	0.16 ± 0.02	0.20 ± 0.03
Σ SFA *p* < 0.001 *	33.81 ± 0.13 ^d^	41.79 ± 0.12 ^b^	36.32 ± 0.14 ^c^	31.57 ± 0.10 ^e^	32.16 ± 0.08 ^e^	43.03 ± 0.11 ^a^	37.85 ± 0.11 ^c^	26.87 ± 0.07 ^f^
Σ MUFA *p* < 0.001 *	23.06 ± 0.11 ^e^	24.92 ± 0.18 ^d^	30.40 ± 0.16 ^b^	33.71 ± 0.16 ^a^	29.55 ± 0.14 ^c^	22.55 ± 0.09 ^e^	25.33 ± 0.12 ^d^	31.14 ± 0.15 ^b^
Σ PUFA *p* < 0.001 *	43.13 ± 0.15 ^a^	33.29 ± 0.12 ^d^	33.27 ± 0.16 ^d^	34.72 ± 0.08 ^c^	38.29 ± 0.09 ^b^	34.42 ± 0.08 ^c^	36.82 ± 0.06 ^b^	41.99 ± 0.11 ^a^
W_L_ (%) *p* < 0.001 *	13.35 ± 0.13 ^c^	13.37 ± 0.13 ^c^	14.06 ± 0.15 ^c^	14.40 ± 0.10 ^c^	29.05 ± 0.09 ^a^	11.84 ± 0.10 ^d^	9.45 ± 0.10 ^e^	7.04 ± 0.10 ^f^
Pr_L_ (g L^−1^ day^−1^) *p* < 0.001 *	0.93 ± 0.01 ^f^	1.54 ± 0.02 ^e^	2.57 ± 0.04 ^d^	6.07 ± 0.06 ^c^	23.30 ± 0.10 ^a^	0.87 ± 0.01 ^f^	1.29 ± 0.02 ^e^	2.07 ± 0.04 ^d^

W_L_ (%)—lipid content in biomass. Pr_L_ (g L^−1^ day^−1^)—lipid productivity. * *p* ≤ 0.05. Means with different letters within a row are significantly different (*p* ≤ 0.05).

**Table 9:** Protein (W_p_; %), carbohydrate (W_c_; %), total polyphenol (TPC; mg GAE g^−1^), and total chlorophyll content (Chl_total_; mg g^−1^) in *D. subspicatus* cultivated on media with different ryegrass enzymatic hydrolysate content.

	BBM	AL1	AL5	AL10	AL25	AL50	AC1	AC5	AC10	AC25
W_p_ (%) *p* < 0.001 *	20.02 ± 0.22 ^a^	16.47 ± 0.14 ^b^	8.34 ± 0.07 ^d^	6.75 ± 0.04 ^e^	5.94 ± 0.04 ^f^	5.79 ± 0.05 ^f^	17.07 ± 0.13 ^b^	9.35 ± 0.08 ^d^	7.76 ± 0.09 ^e^	10.51 ± 0.15 ^c^
W_c_ (%) *p* < 0.001 *	24.71 ± 0.11 ^f^	42.50 ± 0.29 ^a^	35.79 ± 0.21 ^c^	27.84 ± 0.14 ^e^	32.87 ± 0.19 ^d^	33.27 ± 0.22 ^d^	41.08 ± 0.21 ^a^	38.98 ± 0.15 ^b^	30.79 ± 0.15 ^d^	30.78 ± 0.14 ^d^
TPC (mg GAE g^−1^) *p* < 0.001 *	3.23 ± 0.03 ^a^	1.57 ± 0.02 ^d^	1.77 ± 0.02 ^c^	1.13 ± 0.03 ^f^	0.90 ± 0.03 ^g^	1.42 ± 0.02 ^e^	2.48 ± 0.03 ^b^	1.32 ± 0.01 ^e^	2.07 ± 0.04 ^c^	1.21 ± 0.03 ^f^
Chl_total_ (mg g^−1^) *p* < 0.001 *	10.95 ± 0.13 ^a^	5.77 ± 0.07 ^b^	4.63 ± 0.07 ^c^	1.26 ± 0.04 ^e^	1.22 ± 0.03 ^e^	0.87 ± 0.01 ^f^	6.54 ± 0.06 ^b^	4.67 ± 0.05 ^c^	1.21 ± 0.03 ^e^	1.00 ± 0.02 ^f^

* *p* ≤ 0.05. Means with different letters within a row are significantly different (*p* ≤ 0.05).

**Table 10:** Dry matter concentration of the biomass (X; g L^−1^), biomass productivity (Pr_X_; g L^−1^ day^−1^), and carbon source consumption (∆S; %) by *D. subspicatus* cultivated in the stirred-tank photobioreactor at the end of the cultivation period (day 15).

	Photobioreactor
X (g L^−1^)	2.01 ± 0.04
Pr_X_ (g L^−1^ day^−1^)	0.13 ± 0.01
∆S (%) glucose	40.74 ± 0.21
∆S (%) xylose	29.43 ± 0.18
∆S (%) arabinose	38.05 ± 0.24

**Table 11:** Fatty acids profile and lipid productivity (Pr_L_) of *D. subspicatus* cultivated in the stirred-tank photobioreactor.

Fatty Acid	w (%)
C8:0	1.77 ± 0.01
C11:0	0.17 ± 0.00
C14:1 cis 9	0.09 ± 0.01
C15:0	0.11 ± 0.00
C16:0	24.71 ± 5.50
C16:1 cis 9	0.56 ± 0.01
C17:0	2.29 ± 0.09
C17:1 cis 10	1.23 ± 0.53
C18:0	13.96 ± 2.04
C18:1 cis 9	35.48 ± 7.41
C18:2 cis 9, 12	10.54 ± 0.45
C18:3 cis 6, 9, 12	0.72 ± 0.28
C18:3 cis 9,12,15	6.61 ± 1.99
C20:0	0.42 ± 0.07
C20:1 cis 11	0.13 ± 0.07
C22:0	0.31 ± 0.05
C23:0	0.06 ± 0.00
C24:0	0.55 ± 0.21
C24:1 cis 15	0.04 ± 0.00
C22:6 cis 4, 7, 10, 13, 16, 19	7.59 ± 0.04
Σ SFA	44.35 ± 2.49
Σ MUFA	37.73 ± 1.97
Σ PUFA	17.92 ± 1.46
W_L_ (%)	22.05 ± 1.09
Pr_L_ (g L^−1^ day^−1^)	2.96 ± 0.29

W_L_ (%)—lipid content in biomass. Pr_L_ (g L^−1^ day^−1^)—lipid productivity.

**Table 12:** Composition of ryegrass, alkaline-pretreated (Al-PRG), and acid-pretreated ryegrass (Ac-PRG) hydrolysates.

Constituent	w (%)		
	Ryegrass	Al-PRG	Ac-PRG
glucan	36.79	60.31	50.21
xylan	21.07	22.85	13.63
arabinan	7.06	1.12	1.22
ASL	2.05	1.78	1.06
AIL	11.09	n.d.	n.d.
AIL + ash	13.14	8.49	27.34
protein	19.62	n.d.	n.d.
ash	2.69	n.d.	n.d.

ASL—acid-soluble lignin content; AIL—acid-insoluble lignin content; n.d.—not determined.

**Table 13:** Cultivation of *D. subspicatus* under different carbon and nitrogen sources and ryegrass enzymatic hydrolysate concentrations.

Label	Carbon Source	Concentration (g L^−1^)	Label	Nitrogen Source	Carbon/Nitrogen	Label	Pretreatment	Ryegrass Enzymatic Hydrolysate (%)
BBM	-	-	CONTROL	-	-	BBM		-
G5	Glucose	5	NO10	NaNO_3_	10:1	AL1	alkaline	1
G10	Glucose	10	NO30	NaNO_3_	30:1	AL5	alkaline	5
G25	Glucose	25	NO50	NaNO_3_	50:1	AL10	alkaline	10
G50	Glucose	50	YE10	Yeast extract	10:1	AL25	alkaline	25
X5	Xylose	5	YE30	Yeast extract	30:1	AL50	alkaline	50
X10	Xylose	10	YE50	Yeast extract	50:1	AC1	acid	1
X25	Xylose	25				AC5	acid	5
A5	Arabinose	5				AC10	acid	10
A10	Arabinose	10				AC25	acid	25
A25	Arabinose	25						

**Table 14:** GC-FID method parameters for the determination of fatty acid ester composition and concentration.

Parameter	Conditions
Column	ZB-FAME (Zebron), 30 m × 0.25 mm, df 0.20 μm
Detector	FID
Carrier gas/Flow rate	Helium/1.2 mL min^−1^
Temperature program	100 °C, 4 min
	10 °C/min → 140 °C
	3 °C/min → 190 °C
	30 °C/min → 260 °C
	260 °C, 2 min
Injector temperature	250 °C
Detector temperature	260 °C
Split ratio	1:15
Injection volume	2 μL

## Data Availability

The dataset is available on request from the authors.

## Abbreviations

The following abbreviations are used in this manuscript:

C/N	carbon to nitrogen ratio
BBM	Bold’s Basal Medium
OD	optical density
UPLC	ultra-performance liquid chromatography
RID	refractive index detector
GC	gas chromatography
FAME	fatty acid methyl esters
FID	flame ionization detector
Chl_a_	chlorophyll-a
Chl_b_	chlorophyll-b
GAE	gallic acid equivalent
∆S	carbon source consumption
MUFA	monounsaturated fatty acids
SFA	saturated fatty acids
PUFA	polyunsaturated fatty acids
YE	yeast extract
HMF	5-hydroxymethylfurfural

## Appendix A

**Table A1:** Composition of Bold’s Basal Medium (BBM).

Component	Concentration (mol L^−1^)
Sodium nitrate	2.94 × 10^−3^
Magnesium sulfate heptahydrate	3.04 × 10^−4^
Sodium chloride	4.28 × 10^−4^
Dipotassium phosphate	4.31 × 10^−4^
Potassium dihydrogen phosphate	1.29 × 10^−3^
Calcium chloride dihydrate	1.70 × 10^−4^
Zinc sulfate heptahydrate	3.07 × 10^−5^
Manganese(II) chloride tetrahydrate	7.28 × 10^−6^
Molybdenum trioxide	4.93 × 10^−6^
Copper(II) sulfate pentahydrate	6.29 × 10^−6^
Cobalt(II) nitrate hexahydrate	1.68 × 10^−6^
Boric acid	1.85 × 10^−4^
EDTA	1.71 × 10^−4^
Potassium hydroxide	5.53 × 10^−4^
Iron(II) sulfate heptahydrate	1.79 × 10^−5^
Sulfuric acid	1 × 10^−5^
